# Blood Pressure and Oxidative Stress among U.S. Adults Exposed to Lead in Military Environments—A Preliminary Study

**DOI:** 10.3390/diseases6040097

**Published:** 2018-10-27

**Authors:** Emmanuel Obeng-Gyasi, Barnabas Obeng-Gyasi

**Affiliations:** 1 Department of Built Environment, North Carolina Agricultural and Technical State University; Greensboro, NC 27411, USA; 2 Department of Biological Sciences, Purdue University, West Lafayette, IN 47907, USA; bobenggy@purdue.edu

**Keywords:** lead exposure, army lead, military lead, cardiovascular lead, military cardiovascular

## Abstract

In this cross-sectional study, lead exposure among those with a history in military environments was examined. Systolic blood pressure (SBP) and diastolic blood pressure (DBP) are clinical markers of blood pressure, while gamma-glutamyl transferase (GGT) is a marker of oxidative stress. These markers and their effects on those exposed to military environments were explored using National Health and Nutrition Examination Survey (NHANES) data from 2009 to 2016. Mean SBP and GGT were significantly elevated in those exposed to military environments, with a moderately significant association existing between blood lead levels (BLLs) and SBP. More attention must be given to lead exposure in military environments to mitigate the risk of exposure.

## 1. Introduction

Lead is an environmentally persistent inducer of adverse neurological, behavioral, cardiovascular, and other adverse systemic outcomes [1,2]. The negative consequences of lead exposure disproportionately affect children due to children’s behavioral patterns, including their high hand-to-mouth activity, their absorption of a higher percentage of ingested lead as compared to adults, and their underdeveloped blood–brain barrier [3]. In the United States and many other countries, anthropogenic sources of lead are varied and include urban soils that have accumulated lead, lead-based paint, and industrial emissions, among other exposure sources [4]. Due to the significant amount of lead put in the environment from these and other sources, population health is at risk [5].

**Figure 1:**
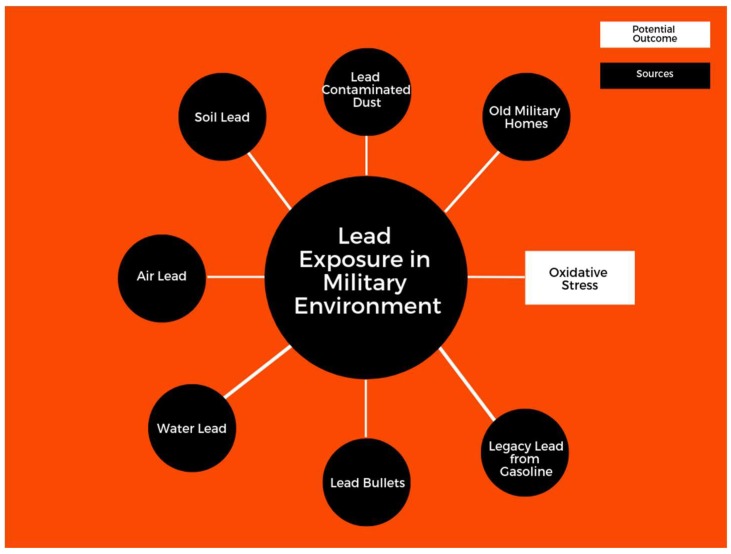
Sources of lead exposure in military environments.

In military environments, the resuspension of dust, vehicular emissions, heavy machinery operations, and oil usage can serve as sources of lead exposure. Shooting with lead-contaminated ammunition is one of the primary sources of lead exposure, as the discharge of lead dust and gases can serve as a source of significant exposure. Blood lead levels (BLLs) in shooters are associated with lead in the air, lead aerosol from guns, the number of bullets fired, and the type of gun used [6]. Figure 1 gives potential sources of lead exposure in military environments.

The consequences of such exposure on adults may include the onset of several developmental [7] and health disorders, including hypertension and cardiovascular diseases [2,8,9]. Oxidative stress potentially serves as an initiating mechanism, inducing damage that can last over the course of a lifetime [10]. These studies and the extensive literature indicate that those with a history in military environments are at elevated risk for lead exposure. In this study, lead exposure among U.S. adults with a military history was examined. The clinical markers for systolic blood pressure (SBP), diastolic blood pressure (DBP), and gamma-glutamyl transferase (GGT) were explored to understand how they manifest in these populations. SBP (the maximum pressure during one heartbeat) and DBP (the minimum pressure between two heart beats) are both markers of blood pressure health. An SBP value of less than or equal to 120 mmHg is considered normal, while an SBP reading of 130 mmHg or more is medically classified as stage I hypertension. An SBP reading of 140 mmHg is considered stage II hypertension. Clinically, a normal DBP reading must be less than 80 mmHg. A DBP reading greater than or equal to 90 mmHg is classified as stage II hypertension. These guidelines, by the American College of Cardiology (ACC) and the American Heart Association (AHA), replaced older guidelines [11] and suggest that adverse blood pressure health starts much earlier than previously thought. GGT, a marker of oxidative stress, is an enzyme that functions in the gamma-glutamyl cycle, catalyzes the transfer of gamma-glutamyl functional groups from molecules such as glutathione, and is found in the liver and other organ tissues, including the kidney and pancreas. Overall, these markers provide an excellent means of exploring the cardiovascular-related health of those who have served in military environments. 

## 2. Materials and Methods

### 2.1. Study Hypothesis

The hypothesis of this study was that those with exposure to military environments would have higher BLLs, blood pressure, and oxidative stress. The objectives of this study were to investigate the effects of lead exposure by analyzing SBP, DBP, and GGT, in addition to examining how various sociodemographic indicators varied between those who had been exposed to military environments and those who had only been exposed to nonmilitary environments. 

### 2.2. Research Design

National Health and Nutrition Examination Survey (NHANES) data from 2009 to 2016 were used to examine the association between lead and cardiovascular-related markers—the SBP, DBP, and GGT of those with a history in military environments. The 2009–2016 NHANES used a representative sample of the U.S. noninstitutionalized population. The blood lead found was analyzed in 1806 people who had served in the military and 16,439 people who had not served in the military. SBP and DBP were analyzed for 2195 people who had served in the military and 20,445 people who had not served. GGT was analyzed for 2277 people who had served in the military and 21,301 people who had not served. In addition, these factors were examined among adults ≥20 years of age in the U.S. noninstitutionalized population in order to see how these factors manifested in a representative sample of U.S. adults. The 2009–2016 datasets were assembled using the publicly available NHANES web tutorial [12]. 

Metal assays in whole blood samples were conducted in the NHANES 2009–2016. In blood samples, metal assays were conducted at the Division of Laboratory Sciences within the National Center for Environmental Health (NCEH) at the Centers for Disease Control and Prevention (CDC). Inductively coupled plasma mass spectrometry (ICP-MS; CDC method No. ITB0001A) via a Perkin-Elmer ELAN^®^ DRC II (PerkinElmer, Norwalk, CT, USA) was used to measure BLLs with the lower limit of detection being 0.07 µg/L. A Beckman Synchron LX20, Beckman UniCel^®^ DxC800 Synchron (Beckman Instruments, Inc., Brea, CA, USA) was used to measure GGT levels using (Collaborative Laboratory Services) in addition to the Roche Modular P chemistry analyzer (Roche Diagnostics, Indianapolis, IN, USA) at the University of Minnesota, Minneapolis. The sample weights, clusters and strata of the sample design was adjusted for using the software Stata SE/15.0 (StataCorp, College Station, TX, USA).

### 2.3. Statistical and Analytical Approaches

Data from adults indicating they had served in the armed forces or had not served in the armed forces, as well as data from the U.S. general adult population, were analyzed in this study. Associations between lead and the markers of interest were analyzed using linear regression. Each exposure–outcome association was explored with its own regression model. Natural log transformation was used after the Shapiro–Wilk test revealed the distribution could be improved. This study adjusted for the covariates of interest (i.e., age, body mass index (BMI), gender, ethnicity, alcohol consumption, and smoking). A *p*-value of 0.05 or less was used to determine significance, with a *p*-value of 0.10 considered to be moderately significant. 

## 3. Results

### 3.1. Sociodemographic and Clinical Markers

In this study, the mean BLL was higher in those who had served in the armed forces than in those who had not. In addition, men represented a larger percentage of those who had served in the armed forces than women. Information on the sociodemographic and clinical makers are presented in Table 1.

### 3.2. Associations between BLLs and SBP, DBP, and GGT in Adults Who Have Served in Military Environments and the U.S. General Adult Population

In this study, there was a positive association between BLLs and SBP, DBP, and GGT in people exposed to military environments, with SBP being moderately statistically significant. Associations between the natural log of BLL (ln *BPb*) and the cardiovascular-related variables are presented in Table 2 below.

In the U.S. general adult population there was a significant association between BLLs, SBP, DBP, and GGT, as presented in Table 3 below. 

## 4. Discussion

### 4.1. Lead and Exposure in Military Environments

Lead sources in military environments are such that those with a history in the military may be exposed to excessive levels [13]. The biologically persistent nature of lead means that such populations may remain continously exposed after leaving these environments. This study of U.S. adults with a potential history of lead exposure in military environments suggests that those with a history in military environments have higher BLLs than those without such a history. In this study, the mean BLL of those who had served in the armed forces was significantly higher than those who had not served. Other exposures in military environments, such as stress, may also have affected the biomarkers in this study. 

In their study of military personnel, Greenberg et al. found that those participating in automatic weapon marksmanship training were at risk of elevated levels of lead exposure. Their study found that during basic training, there was a 95% likelihood that up to 25% of instructors would be exposed to lead above the action level (AL) (25 μg/m^3^). There was also a 99% likelihood that up to 5% of trainees would be exposed. They also found that during advanced training, there was a 90% likelihood that 10% of instructors—and a 99% likelihood that up to 10% of trainees—would be exposed above the AL [14]. This demonstrates that exposure may begin as early as basic training and continue during the entire duration of one’s time in the military in both indoor and outdoor environments.

Rocha et al. conducted a study of the shooting habits of police officers and possible lead exposure prior to a training course. Analysis of BLLs was performed prior to and after the training course. The results indicated that all of the participants had significantly increased BLLs after the course [15]. Similar trainings occur in many military environments, and populations may be exposed in similar ways.

GGT is a marker of oxidative stress, and was found in this study to be more elevated in those who had served in the armed forces than in those who had not served. Epidemiological studies have reliably suggested that serum GGT is a sensitive marker of oxidative stress [16,17]. Associations have also been found between BLLs and GGT in adults [18]. Oxidative stress occurs when reactive oxygen and reactive nitrogen species are produced, which then causes dysfunction in macromolecules such as nucleic acids and proteins [19]. This may speak to the mechanism by which lead induces adverse clinical outcomes in military environments. These adverse clinical outcomes, created by mechanisms such as oxidative stress and inflammation, may contribute to elevated blood pressure.

In this study, the mean SBP was elevated in those who had served in military environments compared to those who had not. In addition, there was a positive association of SBP, DBP, and GGT among those who had served in the armed forces, with SBP being moderately statistically significant. 

In this study, the data of the U.S. general adult population older than 20 years of age indicated that lead was significantly associated with all three biomarkers. This would seem to suggest that lead is less associated with the markers of interest found in those exposed to military environments than with the U.S. population in general. However, the smaller sample size for those exposed to military environments may have affected the results. This is demonstrated by the analysis of larger samples in this study that resulted in significant positive associations with all three markers: this is in contrast to previous studies by Obeng-Gyasi et al. [2] that demonstrated a positive association with DBP, but not SBP, in a smaller sample. Occupational studies on lead and blood pressure have varied, with some showing no difference [20] and others showing differences [2].

This study supports the work of Smoley et al., who, considering hypertension to be 140/90 and prehypertension to be 120–139/80–89, found in a study of military personnel that around 10% of the 15,391 participants were hypertensive and around 60% were prehypertensive [21]. Lead’s general association with blood pressure has been found by other studies [22,23], but the situation in military environments may be worse due to elevated anthropogenic sources of exposure in contrast to the experiences of the general population.

In this study, smoking and alcohol consumption were found to be elevated among those who had served in the armed forces compared to those who had not. In recent studies, tobacco and alcohol use in military environments was found to be elevated, with increases of heavy alcohol use reported [24]. In these environments, deployment status played a role in smoking and the drinking alcohol. According to Smith et al., smoking initiation occurred in 1.3% of nondeployers and 2.3% of deployers. Among those who had smoked previously, they found that 28.7% of nondeployers and 39.4% of deployers resumed smoking. Those who had never smoked who experienced combat during deployment were 1.6 times more likely to begin smoking (95% CI = 1.2, 2.3), while past smokers were 1.3 times more likely to resume smoking than those who had not experienced combat [25]. For these reasons, smoking and alcohol consumption were adjusted for in this study, with adjusted regression analysis still indicating that lead affected cardiovascular-related markers. 

### 4.2. Limitations

BLLs indicate relatively recent exposure, while bone lead gives insight into longer-term exposure. A complete view of the exposure levels to lead would have been achieved if both BLL and bone lead level data were available [26]. In addition, as this is a cross-sectional study, a longitudinal study factoring in exposure during a significant portion of an individual’s life would have offered deeper insight. Finally, the length of military service was unknown, which may have altered levels of exposure. 

## 5. Conclusions

Those with a history of serving in military environments have higher blood lead levels and elevated blood pressure. The increased levels of exposure to lead among these populations is such that they may endure an additional burden compared to those not in the armed forces. This additional burden can accumulate during the life of exposed personnel and induce or further propagate cardiovascular-related pathologies. Larger studies are needed to explore the results here in greater detail. 

## Figures and Tables

**Table 1:** Sociodemographic and clinical markers in the study.

	Served in Armed Forces *N* = 2391	Did Not Serve in Armed Forces *N* = 22,747
Mean blood lead level (BLL) (95% CI)	1.83 (1.68–1.98)	1.33 (1.27–1.38)
Mean age (95% CI)	59.47 (58.52–60.42)	44.64 (44.08–45.21)
Gender percent	Men 91.9% Women 8.10%	Men 43.68% Women 56.32%
Education percent 1 = Less than ninth grade 2 = 9–12th grade—no diploma 3 = High school graduate/GED or equivalent 4 = Some college or associate degree 5 = College graduate or above	1 = 1.89% 2 = 7.57% 3 = 22.69% 4 = 39.40% 5 = 28.43%	1 = 6.08% 2= 11.01% 3 = 21.25% 4 = 31.17% 5 = 30.49%
Ethnicity percent 1 = Mexican-American 2 = Other Hispanic 3 = Non-Hispanic White 4 =Non-Hispanic Black 5 = Other race, including multiracial	1 = 2.88% 2 = 2.71% 3 = 78.09% 4 = 11.34% 5 = 4.98%	1 = 9.50% 2 = 6.29% 3 = 64.08% 4 = 11.64% 5 = 8.48%
Smoking percent (95% CI)	62.91% (59.81–65.90)	41.41% (40.10–42.72)
Alcohol percent (95% CI)	87.44% (85.35–89.27)	75.77% (74.07–77.38)
Systolic blood pressure (SBP)	126.71 (125.67–127.74)	120.69 (120.19–121.18)
Diastolic blood pressure (DBP)	69.44 (68.64–70.23)	69.59 (69.05–70.13)
Gamma-glutamyl transferase (GGT)	31.02 (28.79–33.25)	26.05 (25.42–26.69)

**Table 2:** Associations between BLLs and cardiovascular-related markers of interest in individuals exposed to military environments.

Variables	ln *BPb* Adjusted (95% CI) ^+^	*p*-Value
SBP	0.299 (−0.016, 0.615)	0.063
DBP	0.100 (−0.108, 0.308)	0.339
GGT	0.052 (−0.30, 0.133)	0.208

^+^ SBP, DBP, and GGT adjusted for age, alcohol consumption, smoking, gender, ethnicity, and body mass index (BMI).

**Table 3:** Associations between BLLs and cardiovascular-related markers of interest in the U.S. general adult population.

Variables	ln *BPb* Adjusted (95% CI) ^+^	*p*-Value
SBP	0.238 (0.122, 0.355)	0.0001
DBP	0.132 (0.049, 0.215)	0.002
GGT	0.095 (0.072, 0.118)	0.0001

^+^ SBP, DBP, and GGT adjusted for age, alcohol consumption, smoking, gender, ethnicity and BMI.
